# Improved preoperative risk stratification in endometrial carcinoma patients: external validation of the ENDORISK Bayesian network model in a large population-based case series

**DOI:** 10.1007/s00432-022-04218-4

**Published:** 2022-08-08

**Authors:** Marcel Grube, Casper Reijnen, Peter J. F. Lucas, Frieder Kommoss, Felix K. F. Kommoss, Sara Y. Brucker, Christina B. Walter, Ernst Oberlechner, Bernhard Krämer, Jürgen Andress, Felix Neis, Annette Staebler, Johanna M. A. Pijnenborg, Stefan Kommoss

**Affiliations:** 1 Department of Women’s Health, University Hospital Tuebingen, Calwerstraße 7, 72076 Tuebingen, Germany; 2 Department of Radiation Oncology, Radboud University Medical Center, Geert Grooteplein Zuid 10, 6525 GA Nijmegen, The Netherlands; 3 Department of Data Science, University of Twente, Drienerlolaan 5, 7522 NB Enschede, The Netherlands; 4 Institute of Pathology, Im Medizin Campus Bodensee, Röntgenstraße 2, 88048 Friedrichshafen, Germany; 5 Institute of Pathology, University Hospital Heidelberg, Im Neuenheimer Feld 672, 69120 Heidelberg, Germany; 6 Department of Pathology and Neuropathology, University Hospital Tuebingen, Liebermeisterstraße 8, 72076 Tuebingen, Germany; 7 Department of Obstetrics and Gynaecology, Radboud University Medical Center, Geert Grooteplein Zuid 10, 6525 GA Nijmegen, The Netherlands

**Keywords:** Endometrial carcinoma, Bayesian network, Personalized medicine, Lymph nodes, Risk stratification

## Abstract

**Purpose:**

Preoperative risk stratification of newly diagnosed endometrial carcinoma (EC) patients has been hindered by only moderate prediction performance for many years. Recently ENDORISK, a Bayesian network model, showed high predictive performance. It was the aim of this study to validate ENDORISK by applying the model to a population-based case series of EC patients.

**Methods:**

ENDORISK was applied to a retrospective cohort of women surgically treated for EC from 2003 to 2013. Prediction accuracy for LNM as well as 5-year DSS was investigated. The model’s overall performance was quantified by the Brier score, discriminative performance by area under the curve (AUC).

**Results:**

A complete dataset was evaluable from 247 patients. 78.1% cases were endometrioid histotype. The majority of patients (*n* = 156;63.2%) had stage IA disease. Overall, positive lymph nodes were found in 20 (8.1%) patients. Using ENDORISK predicted probabilities, most (*n* = 156;63.2%) patients have been assigned to low or very low risk group with a false-negative rate of 0.6%.

AUC for LNM prediction was 0.851 [95% confidence interval (CI) 0.761–0.941] with a Brier score of 0.06. For 5-year DSS the AUC was 0.698 (95% CI 0.595–0.800) as Brier score has been calculated 0.09.

**Conclusions:**

We were able to successfully validate ENDORISK for prediction of LNM and 5-year DSS. Next steps will now have to focus on ENDORISK performance in daily clinical practice. In addition, incorporating TCGA-derived molecular subtypes will be of key importance for future extended use. This study may support further promoting of data-based decision-making tools for personalized treatment of EC.

## Introduction

Individualized endometrial carcinoma (EC) care has been introduced many years ago by defining the adjuvant therapy after upfront surgery based upon patient-specific risk stratification (Concin et al. 2021).

After Bokhman et al. had established the Type I and II concept for EC in the early 1980s (Bokhman 1983), postoperative treatment decisions were almost exclusively made according to histopathological features such as histological subtype, grade and depth of myometrial invasion.

More recently, the Cancer Genome Atlas (Huvila, Pors et al. 2021) endometrial collaborative project identified four distinct molecular EC subtypes (Kandoth et al. 2013). The prognostic value of this new molecular-driven approach has been confirmed in many studies since then (Talhouk et al. 2017; Kommoss et al. 2018, León-Castillo, de Boer et al. 2020). Today the scientific community widely agrees that these new findings will help to improve personalized medicine and treatment guidelines have already incorporated the option to take molecular classification into account when considering adjuvant therapy (Vermij et al. 2020; Concin et al. 2021).

While TCGA-derived parameters including *POLE* mutation status, mismatch repair (MMR) status and p53 immunhistochemistry (IHC) may be obtained from curettage specimens, for final risk stratification tumor stage and lymphovascular space invasion (LVSI) are relevant (Concin et al. 2021). Patients with abnormal p53 have the worst outcome and often present with high-grade and advanced stage disease.

Thus, current risk models are of limited help in guiding preoperative patient counselling. Moreover, it is well known that preoperative histopathological parameters like histotype and grade have been shown to have only moderate reproducibility with the final tumor specimen (Clarke and Gilks 2010; Gilks et al. 2013; Han et al. 2013).

While radical procedures such as systematic pelvic and paraaortic lymphadenectomy or radical hysterectomy were considered standard procedures in EC treatment for many years (Vitale et al. 2016; Brooks et al. 2019), surgeons tend to apply less invasive techniques today (Gasparri et al. 2019). According to current literature, surgical lymph node assessment is considered unnecessary for the majority of patients and pelvic sentinel node biopsy may be adequate even for patients presenting with high-risk features, if applied according to the NCCN guidelines (Kitchener et al. 2009, Bodurtha Smith et al. 2017, Frost, Webster et al. 2017, Gasparri et al. 2019). However, thorough lymph node sampling may still be crucial for patients at high risk for distant tumor spread or in case sentinel node detection fails (Frost, Webster et al. 2017). In the absence of sufficient preoperative risk stratification, there has been a call from the gynecologic oncology community to develop new tools supporting preoperative surgical decision making. It is the overall goal to prevent patients from surgical morbidity without withholding decisive procedures necessary to tailor adjuvant treatment.

Taking the latter issues into account Reijnen et al. recently developed an easy-to-use preoperative risk stratification tool termed “ENDORISK” (Reijnen et al. 2020), a Bayesian Network model (Lucas, van der Gaag et al. 2004). Using a small number of clinical and immunohistochemical parameters (p53, ER/PR and L1CAM) available after the initial diagnosis, ENDORISK can predict the risk of lymph node metastasis (LNM) as one of the most important prognostic factors for poor outcome and 5-year disease-specific survival (DSS). According to the ENDORISK risk estimation, patients with high-risk for LNM may be subjected to more radical surgical procedures. Vice versa surgical lymph node assessment may be omitted in patients at low risk for LNM (Reijnen et al. 2020).

According to the Institute of Medicine guidelines for the development of Omics-based tests, it was the aim of this study to externally validate initial findings reported after ENDORSIK was applied to a discovery and confirmation cohort. Herein we present ENDORISK performance in a large population-based cohort including consecutive endometrial carcinoma patients from an independent institution.

## Methods

### Patient cohort

Patients treated for primary EC between 2003 and 13 of any stage and histotype were identified from the Tübingen University Women’s Hospital patient records. Relevant parameters were collected through a retrospective chart review, histopathological review diagnoses were available for all patients. The study was approved by the ethics committee of the University of Tuebingen.

Following the ENDORISK minimal requirements, all patients had to meet the following minimal inclusion criteria:

- Histologically diagnosed endometrial carcinoma of any histological subtype
- Histopathological examination of lymph nodes
- Availability of 5-year DSS
- Availability of pre-operative tumor grade
- Availability of at least three out of four immunohistochemical biomarkers:
  - L1 cell adhesion molecule (L1CAM) status (< 10%, ≥ 10%—membranous staining)
  - Estrogen receptor (ER) status (< 10% negative, ≥ 10% positive—nuclear staining)
  - Progesterone receptor (PR) status (< 10% negative, ≥ 10% positive—nuclear staining)
  - Immunohistochemical p53 status (wildtype, complete absence, overexpression, cytoplasmatic)
- Availability of at least one of four preoperative clinical markers:
  - Atypical endometrial cells present in PAP-smear (No, Yes)
  - Suspected lymph node ≥ 10 mm short axis diameter in CT-scan (No, Yes)
  - Cancer antigen 125 [CA125] (< 35 kU/l, ≥ 35 kU/l)
  - Platelet count (< 400 Tsd/µl, ≥ 400 Tsd/µl)

Preference was given to data available from curettage specimens, if immunohistochemical staining result of curettage and hysterectomy samples were discordant, i.e., negative and positive, final results were considered as positive.

### Statistical analysis

An exploratory analysis of possible selection bias of the study population was performed by the w2-test. After, ENDORISK was applied to our cohort according to the methods previously described by Reijnen et al. (2020). Lymph node metastasis and 5-year survival prediction were calculated using the GeNie Academic software (Version 3.0, BayesFusion) for each case. To validate ENDORISK, the overall performance of the prediction model was evaluated by calculating Brier scores [mean squared difference between predicted probability and observed outcome, between 0 and 1; lower Brier score indicates better accuracy of the probabilistic predictions (Reijnen et al. 2020)]. Discrimination was assessed using a receiver operating characteristic curve (ROC) generated by plotting sensitivity against 1-specificity. To quantify the discriminative performance areas under the curves (AUC) were calculated. Finally, the predicted number of events were compared to the observed outcome. Calibration curves were plotted. All exercises were performed for lymph node metastasis and 5-year DSS prediction separately.

For calculations and statistical analysis, different R-Scripts (R Studio, RStudio, Inc., Boston, MA, USA) were used.

## Results

### Patient characteristics

A total of *n* = 450 patients were identified, of which *n* = 247 (54.9%) met the inclusion criteria as given above. Data on race and ethnicity of patients were not available as it was not recorded. No significant selection bias was found after exploration analysis of patient characteristics (patient age, histology, grade, stage) comparing patients with and without available minimal dataset. Median age was 64 years (range: 33–90, Table 1). A majority of 156 (63.2%) patients was diagnosed with FIGO stage IA disease, of the remaining cases 52 (21.1%) were stage IB, 12 (4.9%) were stage II and 27 (10.9%) were stage III/IV. Histological type was found to be endometrioid in *n* = 193 (78.1%), serous in *n* = 19 (7.7%), mucinous *n* = 19 (7.7%) and clear cell in *n* = 5 (2.0%) cases. Preoperative grade distribution included 87 (35.2%) grade 1, 106 (42.9%) grade 2 and 54 (21.9%) grade 3. Lymph node metastasis has been observed in *n* = 20 (8.1%) cases.

**Table 1:** Clinical parameters of ENDORISK validation cohort (L1CAM—L1-cell adhesion molecule, membranous staining— < 10% negative/ ≥ 10% positive, ER nuclear staining— < 10% negative/ ≥ 10% positive, PR nuclear staining— < 10% negative/ ≥ 10% positive)

	ENDORISK validation cohort
Patients, *n* (%)	247 (100.0)
Median age (range)	64 (33–90)
FIGO stage, *n* (%)	
IA	156 (63.2)
IB	52 (21.1)
II	12 (4.9)
III/IV	27 (10.9)
Histotype, *n* (%)	
Endometrioid	193 (78.1)
Serous	19 (7.7)
Mucinous	19 (7.7)
Clear Cell	5 (2.0)
Other	11 (4.5)
Preoperative Grade, *n* (%)	
G1	87 (35.2)
G2	106 (42.9)
G3	54 (21.9)
Estrogen receptor (ER), *n* (%)	
Positive	219 (88.7)
Negative	25 (10.1)
Unknown	3 (1.2)
Progesterone receptor (PR), *n* (%)	
Positive	202 (81.8)
Negative	45 (18.2)
Unknown	0 (0.0)
p53 immunohistochemistry, *n* (%)	
Wildtype	209 (84.6)
Abnormal	38 (15.4)
Unknown	0 (0.0)
L1CAM, *n* (%)	
Positive	55 (22.3)
Negative	192 (77.7)
Unknown	0 (0.0)
Median CA125 level, kU/l (range)	14 (2–478)
Median platelet count, Tsd/µl (range)	283 (116–949)
Median follow-up time, months (range)	101 (5–191)

Median follow-up time of our cohort was 101 months (range: 5–191 months). A total of 32 DSS events were observed.

### Validation—lymph node metastasis prediction

**Fig. 1:**
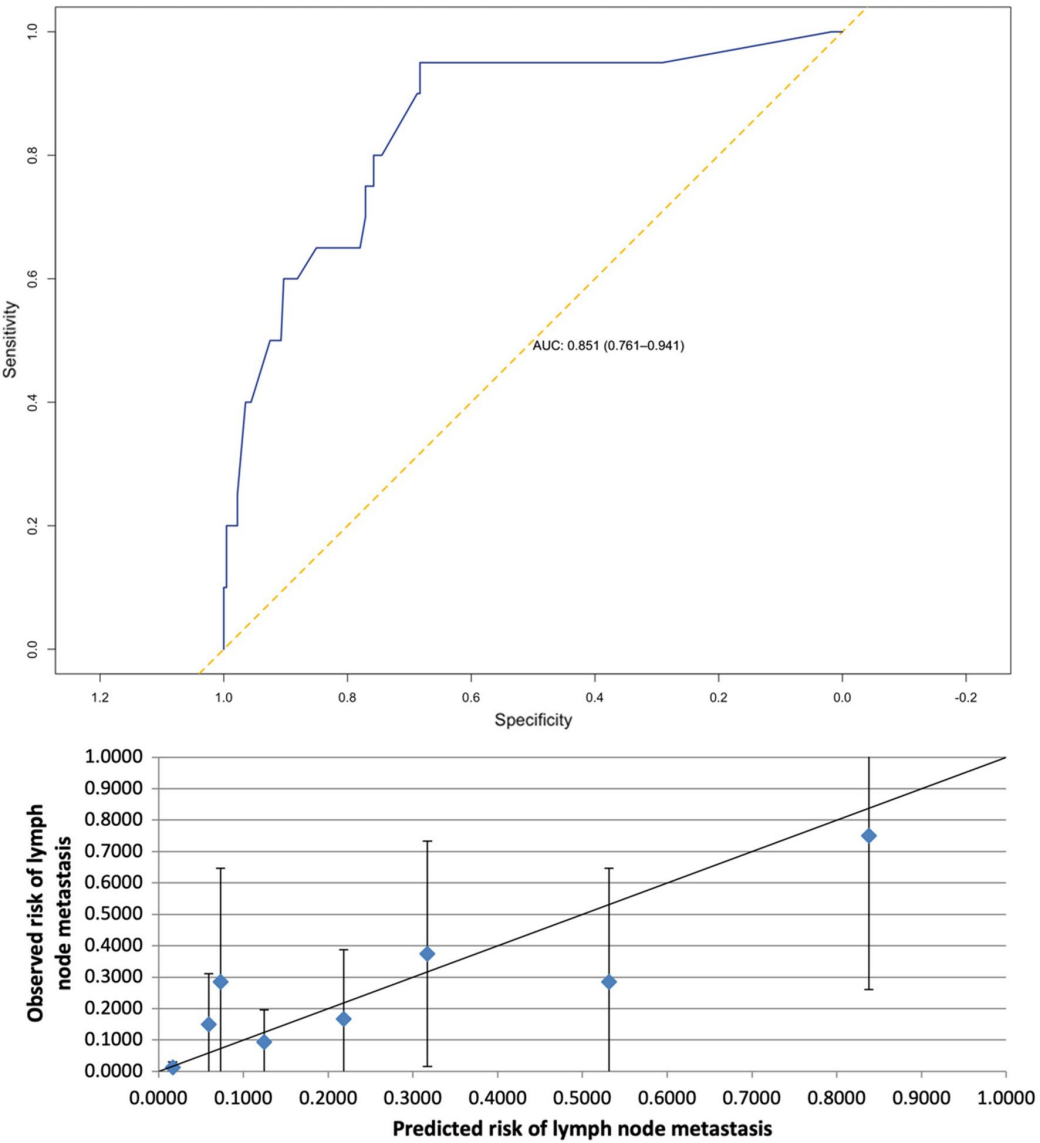
ROC and AUC and Calibration plot for prediction of risk of LNM (vertical bars represent 95% CI, *ROC* receiver operating characteristic, *AUC* area under the Curve, *LNM* lymph node metastasis, *CI* confidence interval)

The AUC for LNM prediction was 0.851 [95% confidence interval (CI) 0.761–0.941, Fig. 1] with a Brier score of 0.06 (Table 2). The ratio of predicted/observed events for LNM was 1.01 (95% CI 0.89–1.13, Table 2). Calibration for risk of LNM is visualized in Fig. 1.

**Table 2:** Performance data ENDORISK (LNM—lymph node metastasis, 5-year DSS—5-year disease-specific survival, AUC—area under the curve, CI—confidence interval)

	LNM	5-year DSS
AUC (95% CI)	0.851 (0.761–0.941)	0.698 (0.595–0.800)
Brier score	0.06	0.09
Predicted *n* (%)	21 (8.5)	228 (92.3)
Observed *n* (%)	20 (8.1)	220 (89.1)
Predicted/Observed ratio (95% CI)	1.01 (0.89–1.13)	1.04 (0.91–1.09)

In 156 (63.2%) patients, ENDORISK prediction of LNM was lower than or equal to 5% (Table 3). The false-negative rate was found to be as low as 0.6% (Table 4), the false-positive rate was 81.2% for LNM prediction, respectively.

**Table 3:** Assigned risk groups based on predicted probabilities of LNM by the ENDORISK Bayesian network (LNM—lymph node metastasis)

Predicted probability of LNM	Risk group	Patients assigned to risk group, *n* (%)	Observed prevalence of LNM, *n* (%)
< 1%	Very low	67/247 (27.1)	1/67 (1.5)
1–5%	Low	89/247 (36.0)	0/89 (0.0)
6–15%	Intermediate	57/247 (23.1)	7/57 (12.3)
16–25%	High-intermediate	17/247 (6.9)	4/17 (23.5)
> 25%	High	17/247 (6.9)	8/17 (47.1)

**Table 4:** Diagnostic accuracy values for the prediction of lymph node metastasis in the validation cohort (PPV—positive predictive value; NPV—negative predictive value; FNR—false-negative rate)

Cut-Off	Sensitivity	Specifity	PPV	NPV	FNR (%)
1%	0.95	0.29	0.11	0.99	1/67 (1.5)
5%	0.95	0.64	0.19	0.99	1/156 (0.6)
10%	0.65	0.81	0.23	0.96	7/190 (3.7)
15%	0.60	0.90	0.35	0.96	8/213 (3.8)
20%	0.50	0.93	0.37	0.95	10/220 (4.5)
25%	0.40	0.96	0.47	0.95	12/230 (5.2)

### Validation—5-year DSS

**Fig. 2:**
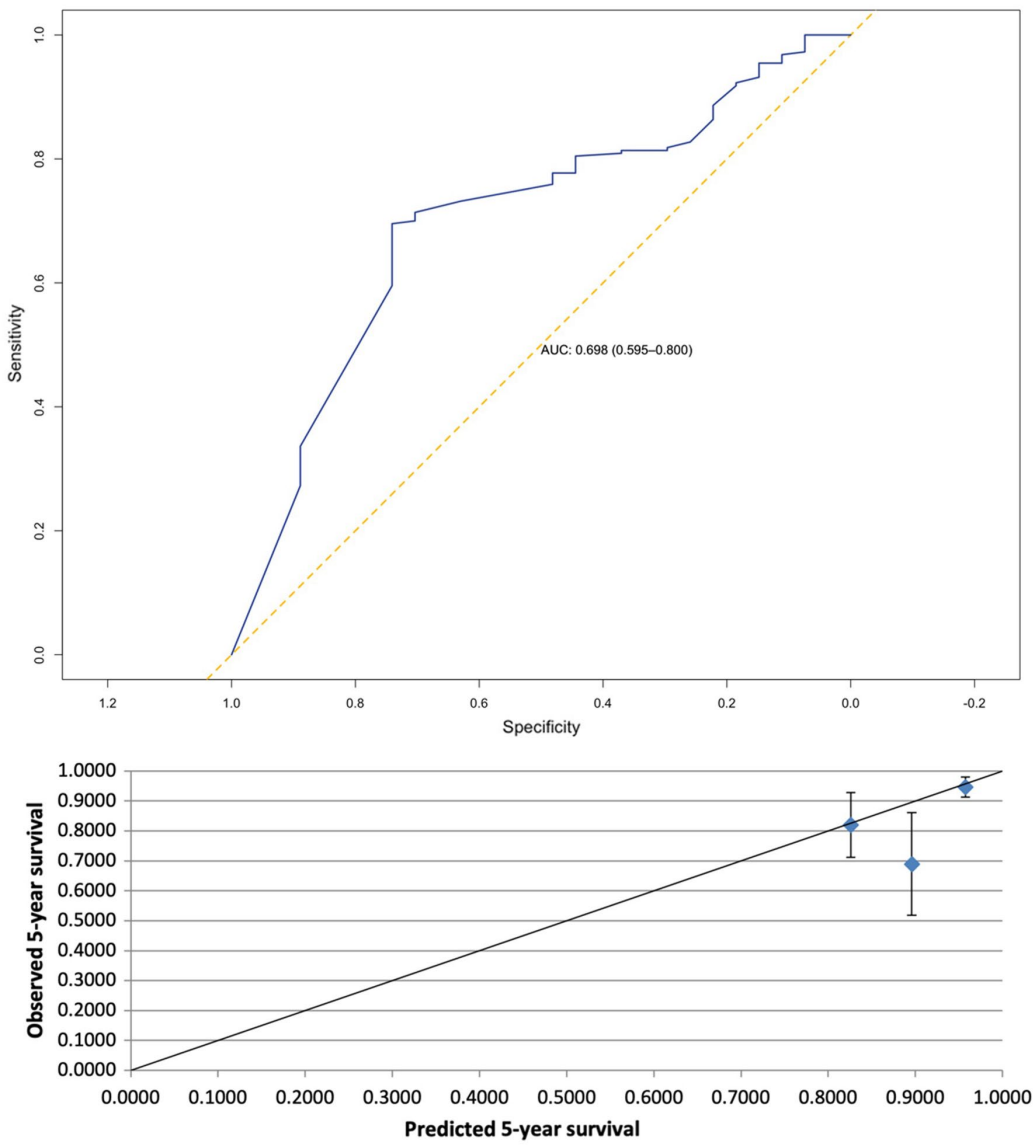
ROC and AUC and Calibration plot for prediction of risk of 5-year DSS (vertical bars represent 95% CI, *ROC* receiver operating characteristic, *AUC* area under the curve, *DSS* disease specific survival, *CI* confidence interval)

The AUC for 5-year DSS prediction was 0.698 [95% confidence interval (CI) 0.595–0.800, Fig. 2] with a Brier score of 0.09 (Table 2). The ratio of predicted/observed events for 5-year DSS was 1.04 (95% CI 0.91–1.09, Table 2). Calibration plot for 5-year DSS is depicted in Fig. 2.

In 105 (42.5%) patients, ENDORISK prediction of a 5-year DSS event was higher than 5%. The false-negative rate was found to be 25.9%, the false-positive rate was 38.6% for 5-year DSS prediction, respectively.

## Discussion

In the present study, we evaluated the ENDORISK preoperative risk prediction model in a large population-based study cohort of consecutive EC patients and were able to demonstrate a high diagnostic performance with an AUC of 0.851 by using this Bayesian network model.

Personalized surgical endometrial carcinoma treatment is currently hindered by poor reproducibility of parameters used for risk classification in the preoperative setting (Gilks et al. 2013). In the light of recent advantages, most importantly the introduction of TCGA-based risk classification, the use of new prediction models will lead to innovative and patient-centered treatment strategies. The scientific community widely agrees that surgical and adjuvant overtreatment with avoidable morbidity occurs in many patients; however, unexpected fatal outcome may be caused by underestimating risk in a significant number of patients at the same time (Concin et al. 2021).

While molecular-based informed decision-making and new prediction models can already be found in the adjuvant setting, surgical strategies are still highly dependent on parameters that are either not available preoperatively or prone to high interobserver variability. Thus, a more personalized treatment approach may help to avoid surgery-associated morbidity in the future. EC patients commonly have multiple comorbidities such as obesity, diabetes and hypertension (Shaw et al. 2016; Moore and Brewer 2017) associated with increased peri- and postoperative complications (Morice et al. 2016). Dedicated preoperative risk models may help to limit more radical procedures to patients at high risk for LNM or distant tumor spread as still the majority of EC patients has favorable outcome by simple hysterectomy and bilateral salpingoophorectomy only (REF). Moreover, limited surgical resources and increasing burdens on healthcare systems will require meticulous patient selection in the future. Time and resource consuming surgical treatments will undergo critical review and there may be a limitation to dedicated centres and specific patient cohorts.

It is well-known that routine lymphadenectomy in clinical early-stage endometrial cancer has not resulted in improved patient outcome and is associated with increased peri- and postoperative morbidity (Frost, Webster et al. 2017). This underlines the relevance of predictive models helping to identify patients that benefit most from more radical surgery.

Current concepts and guidelines for endometrial carcinoma patient care include the use of sentinel biopsy procedures. While such surgical equipment is not generally available, surgeons might still be faced with technical issues. Failed detection or questionable intraoperative findings may require ad hoc decisions whether a systematic lymph node dissection is still necessary. The use of reliable LNM prediction tools might therefore not only be restricted to preoperative patient counselling. While intraoperative pathologic examination on frozen section samples is available to almost any dedicated gynecological oncology center, there are still many patients that undergo endometrial carcinoma surgery in rather small hospitals or local healthcare settings, and definitive risk-classification will be not available until the final pathology report is made for those patients. ENDORISK may help select patients with substantial risk of LNM preoperatively to refer to gynecological oncology centers.

It was recently shown that the ENDORISK Bayesian Network model can help physicians to decide whether lymph node removal is necessary or can be omitted due to lack of patient benefit (Reijnen et al. 2020).

With this validation cohort of 247 consecutive endometrial carcinoma patients, we were able to externally validate the ENDORISK Bayesian network for prediction of LNM risk and 5-year DSS. A Brier score of 0.06 has confirmed a good overall performance of the model in our cohort, with a lower Brier score indicating a better accuracy of the probabilistic predictions. The ENDORISK discriminative performance for LNM prediction (AUC 0.851) was shown to be even higher as compared to the original discovery cohort published by Reijnen et al. (2020).

In terms of 5-year DSS prediction, ENDORISK performance was found to be not quite as good in our validation series as the model seems to overestimate patients’ survival outcomes. This part of the ENDORISK network might be further improved by integration of molecular profiling to refine outcome in relation to adjuvant therapy and both clinicopathological and TCGA data. Nevertheless, we can still report an adequate discriminative performance (AUC 0.698) and again a low Brier score (0.09).

In summary, we were able to retrospectively apply ENDORISK to a large series of patients indicating that ENDORISK may aid informed decision-making in the future. Furthermore, our analysis confirmed that the majority of patients suffering from EC can be classified as (very) low risk for LNM (63.2%, false-negative rate 0.6%). These findings reinforce that systematic lymphadenectomy or sentinelnode procedure can be safely omitted in a large number of patients.

The ENDORISK validation exercise presented herein has some limitations. Due to its retrospective manner, almost 50% of patients diagnosed with endometrial carcinoma between 2003 and 13 at our hospital had to be excluded because of missing data. Immunohistochemical scores were obtained retrospectively using a tissue microarray which was built from diagnostic and hysterectomy samples.

Until today ENDORISK was applied to a total of 1077 patients, of which the vast majority (*n* = 867; 81%) was diagnosed with endometrioid adenocarcinoma. It remains a matter of speculation if rare types of endometrial carcinoma (serous, clear cell, mucinous, carcinosarcomas) may be underrepresented, potentially biasing study results. In addition, poor-reproducibility was reported for high-grade subtypes of endometrial carcinoma (grade 3 endometrioid, serous, clear cell, or carcinosarcoma) (Gilks et al. 2013), potentially leading to incorrect histotyping and grading in a significant number of cases used for discovery and validation purposes. Thus, it might be desirable to limit modeling parameters to highly objective data only if therapeutic decisions are to be made. Therefore, next steps will have to focus on implementing TCGA-derived molecular information which can also be generated from preoperative samplings. Molecular-based classification is known to provide highly prognostic as well as predictive information (Talhouk et al. 2017; Kommoss et al. 2018) and might help to make preoperative prediction models even more robust. Looking at current trends and future perspectives in endometrial carcinoma treatment TCGA-based risk classification seems to be essential, ongoing collaborative research efforts will thus have to focus on implementing appropriate surrogate parameters.

Our study shows that preoperative risk models such as ENDORISK Bayesian network can be a valuable tool in daily clinical decision making. However, future trials will have to show how preoperative prediction models can finally guide surgical treatment in order to ultimately improve endometrial carcinoma patient outcome.
